# MIF and MMP-9 Serum Changes in Type II Diabetes and Non-Diabetic Subjects: A Short Communication

**DOI:** 10.30699/IJP.2021.131429.2456

**Published:** 2021-07-06

**Authors:** Alireza Rastgoo Haghi, Nasrin Khorami, Mahtab Fotoohi, Abbas Moradi

**Affiliations:** 1 *Department of Pathology, School of Medicine, Hamadan University of Medical Sciences, Hamadan, Iran*; 2 *Department of Internal Medicine-Endocrinology, School of Medicine, Hamadan University of Medical Sciences, Hamadan, Iran *; 3 *Department of Community Medicine. Hamedan University of Medical science. Iran*

**Keywords:** Diabetes Type 2, HbA1C, MIF, MMP-9

## Abstract

**Background & Objective:**

Diabetes is a metabolic disease and is associated with failure of various organs. Macrophage migration factor (MIF) and matrix metalloproteinase (MMP-9) are two of the most important factors in the pathogenesis of diabetes.

**Methods:**

In this descriptive-analytical study, 30 patients with type 2 diabetes mellitus from Hamadan Diabetes Center were selected by convenience sampling. Moreover, 30 healthy first-degree relatives and 30 unrelated non-diabetics, were examined for MMF and MMP-9 and their variations based on age, gender, body mass index (BMI) and hemoglobin A1C.

**Results & Conclusion:**

The mean and standard deviation of MIF in diabetic patients, and relatives and non-relatives of diabetic patients were 592.87±78.19, 131.82±88.27 and 94.63±23.88, respectively (*P*<0.001). The mean and standard deviation of the MMP-9 in diabetic patients, and relatives and non-relatives of diabetic patients were 2570.64±2220.03, 918.57±650.08 and 629.09±288.32, respectively (*P*<0.001). MIF and MMP-9 did not have a significant relationship with age, sex, duration of disease and BMI. However, we observed a direct and significant correlation between hemoglobin A1C and the level of MIF and MMP-9 (*P*<0.001). In patients with type 2 diabetes, serum levels of MMP-9 and MIF, consistent with HbA1c, increase with no significant association with age, sex, BMI and duration of diabetes.

## Introduction

Diabetes mellitus is a metabolic and multifactorial disease characterized by hyperglycemia, and accompanied by various organ failures (1, 2). Studies on inflammatory cytokines that cause diabetes are of great importance. Macrophage migration factor (MIF) and matrix metalloproteinase (MMP-9) are the most important factors in the pathogenesis of diabetes (3,4). MIF affects the expression of Toll-like receptor (TLR-4) on antigen presenting cells, preserving the ability of macrophage to interfere with P53 inhibition and has tautomerase and oxidoreductase activity (5-7). Studies on the animal models have shown that inhibition of MIF as a therapeutic approach. Another group of factors affecting the pathogenesis of diabetes and the associated complications are MMPs (8). Hyper-glycemic conditions increase the expression of MMP-1, MMP-2 and MMP-9 genes and increase their activity in endothelial cells and macrophages (9-11). The present study evaluates the levels of MIF and MMP-9 in non-diabetics in the families with family members who have type 2 diabetes mellitus. This provides promising evidence. 

## Material and Methods

In this study, 30 patients with type 2 diabetes, 30 non-diabetics selected from first-degree relatives of patients with diabetes and 30 unrelated non-diabetics as control group were selected. All subjects were interviewed and serum samples were prepared for Fasting Blood sugar (FBS) and HbA1c tests, and were approved according to the previous medical histories and the results of the healthy group. Diabetics were categorized based on HbA1c level into optimal control group (HbA1c<7) and undesirable control group (HbA1c>7). MMP-9 and MIF levels were measured by direct sandwich ELISA (East biopharm Company kit) according to the manufacturer's instructions as follows: 40 μL of the sample was mixed with 10 μL of MMP-9 and MIF antibodies and 50 μL of Streptavidin-HRP. Then, the lid was tightly closed. The plate was shaken slowly and incubated at 37°C for 60 minutes. The sample was then diluted 30 times and washed with water. Subsequently, we added 50 μL of colored solution A and B to each of the wells. We slowly mixed and incubated the plate for 10 minutes at 37°C. Then, 50 μL of stop solution was added to each of the wells to stop the reaction. At this point, the color quickly changed from blue to yellow. The device was zeroed at 450 nanometers (nm) at a wavelength of 450 nm and the sample standard OD was accurately measured and then, the final concentration of the sample was obtained. The measurement range of the test kit for testing MIF was between 10-30 ng/mL. The sensitivity of the kit was 54.6 ng/ mL, and for MMP-9 in the range of 30-35 ng/mL, with a sensitivity of 15.12 ng/L. 

## Results & Discussion

Based on Kruskal-Wallis test and Tukey's post hoc test, there was a significant difference in the mean level of macrophage migration inhibitor in type 2 diabetic patients and non-familial control group (*P*<0.05). However, the difference between the mean levels of the inhibitory factor of macrophage migration in the control group was not statistically significant (Table 1). Also, based on Kruskal-Wallis non-parametric test and Tukey's post hoc test, the mean level of MMP-9 in type 2 diabetic patients was significantly more than familial and non-familial control groups (*P*<0.05). However, the difference between the mean level of MMP-9 in the familial and non-familial control group was not statistically significant (Table 1). Moreover, there was no statistically significant difference between the male and female sexes in terms of the mean level of inhibitory factor of macrophage migration and also the MMP-9 level (Table 2). Results of the Pearson correlation test showed a reverse correlation between age and level of macrophage migration inhibitor (r=-0.218, *P*=0.247) and MMP-9 (r=-0.279, *P*=0.136), but it was not statistically significant. There was a direct and significant correlation between hemoglobin A1c and the level of macrophage migration inhibitor (r=0.821, *P*<0.001) and MMP-9 (r=0.745, *P*<0.001). There was no significant correlation between the duration of type 2 diabetes mellitus and the level of macrophage migration inhibitor (r=0.165, *P*=0.385) and MMP-9 (r=0.196, *P*=0.299). There was no significant correlation between BMI and Type 2 diabetes mellitus with macrophage migration inhibitor (r=-0.242, *P*=0.197) and MMP-9 (r=-0.262, *P*=0.163). By increasing the hemoglobin A1C, the mean MIF and MMP-9 also increased significantly (Table 3).

**Table 1:** Mean of factor levels in different study groups

Groups	Diabetics	First degree family	non-relatives	P-value
Factor levels (Mean ± SD)				
MIF	592.78 ± 678.19	131.82 ± 88.27	94.63 ± 23.88	<0.001
MMP-9	2570.64 ± 2220.03	918.57 ± 650.08	629.09 ± 288.32	<0.001

**Table 2:** Mean of factor levels in different genders of the study

Factor levels in different genders		Mean ± Standard Deviation	P-value^*^
MIF	**Male**	712.42 ± 754.21	0.344
	**Female**	532.96 ± 694.05	
MMP-9	**Male**	2864.79 ± 2377.45	0.379
	**Female**	2423.56 **± **2185.60	

^*^ Non-parametric Mann-Whitney test

**Table 3:** Comparison between the factor levels and the different levels of HbA1c

Factors and HbA1c levels		N	Mean ± SD	95% CI		P-value
				Upper Bound	Lower Bound	
MIF	**4.2-6.9**	75	136.364 **± **98.277	158.9766	113.7533	<0.001
	**7-8.9**	12	677.529 **± **530.514	1014.6021	340.4562	
	**9 ≤**	3	2073.100 **± **807.904	4080.0456	66.1544	
	**total**	90	273.078 **± **452.223	367.7944	178.3616	
MMP-9	**4.2-6.9**	75	877.485**±**638.029	1024.2830	730.6885	<0.001
	**7-8.9**	12	3095.390**±**1831.426	4259.0239	1931.7577	
	**9 ≤**	3	6864.200**±**2440.418	12926.5358	801.8642	
	**total**	90	1372.763**±**1584.351	1040.9275	1584.35172	

In the present study, the mean MIF and MMP-9 level in type 2 diabetes mellitus patients was significantly higher than that of non-familial control group. However, there was no significant difference between the control group in diabetic families and the non-related control group in terms of MIF and MMP-9 levels. The results of Yuriko *et al.* showed that MIF is an effective polytropic molecule of pancreatic islets and pro-inflammatory cytokines that not only plays a role in diabetes, but also in the early stages of the disease and its risk factors such as obesity (12). In the study of Lee *et al.,* The results showed that MMP-9 plasma levels were significantly lower in diabetic patients than in control group (13). Similarly, these results repeated in the study of Lewandowski *et al*.. There was a reverse correlation between BMI and MMP-9 levels, and there was a positive correlation between HbA1c and MMP-9 levels (14). Comparing metalloproteinase 2 and 9 and tissue inhibitors in diabetic and non-diabetic patients, Derosa *et al.* showed that MMP-9 plasma levels were significantly higher in patients with diabetes than in healthy subjects (15). Moreover, in a study conducted in India regarding MMP in type 2 diabetic patients, the results showed that MMP levels in type 2 diabetic patients increased significantly (8). The results of this study were consistent with the studies of Lewandowski, Derosa and Das *et al.* in terms of the increase in serum MMP levels in diabetic patients. Our results were inconsistent with the results of Lee *et al. *(13-15). In the present study, there was no significant difference between gender and the mean level of macrophage migration inhibitor and MMP-9 in type 2 diabetes. There was a significant and direct correlation between age, BMI, duration of disease and hemoglobin A1C and macrophage migration inhibitor and MMP-9 levels. However, only the correlation between hemoglobin A1C and the level of macrophage migration and MMP-9 inhibitor was statistically significant. In the study of Lewandowski *et al*., there was a reverse correlation between BMI and MMP-9 levels, and a positive correlation between hemoglobin A1c and MMP-9 level (14). These results were similar to our study findings. Considering that in diabetic patients, MMP-9 and MIF levels are elevated, and are directly related to hemoglobin A1c levels, it is suggested to use both of these factors to measure the presence of diabetes, based on the existing conditions and possibilities. It is suggested that in an analytical study, the role of MMP-9 and MIF factors in the incidence of diabetes complications should be investigated. In order to increase the reliability of the study, it is recommended to repeat the study in a multicenter study with a larger sample size.

## Conclusion

In diabetics, MMP-9 and MIF levels were consistent with the level of hemoglobin A1C, but there was no significant increase in MMP-9 and MIF in first-degree relatives of diabetic and normal people. Since these factors did not significantly increase in diabetics and their normal relatives, they cannot be used as predictors of diabetes.
